# Supplementary material for: Aggregation Behavior, Antibacterial Activity and Biocompatibility of Catanionic Assemblies Based on Amino Acid-Derived Surfactants
Source: Int J Mol Sci. 2020 Nov 24;21(23):8912. doi: 10.3390/ijms21238912 (PMC7727793; doi:10.3390/ijms21238912)
Supplement: Supplementary file 1 [file ijms-21-08912-s001.pdf]

SUPPLEMENTARY MATERIALS

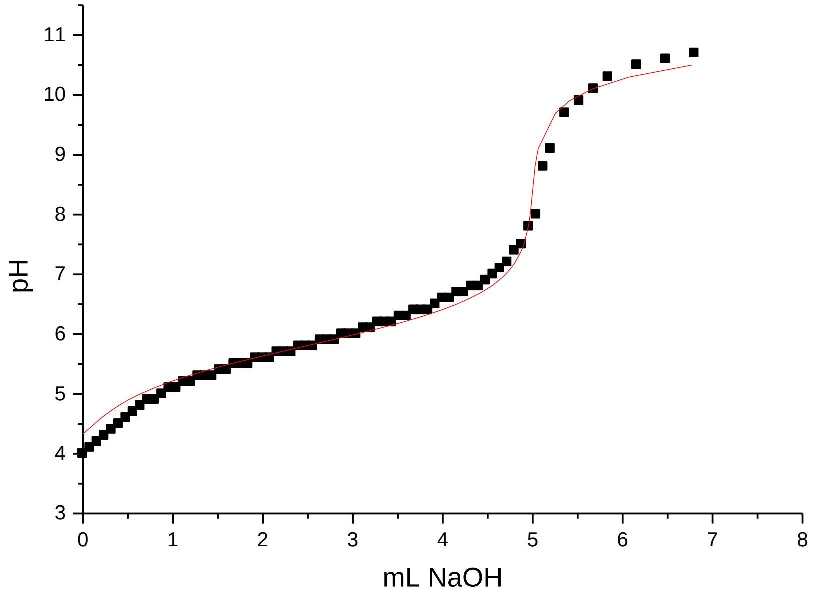

Figure S1. Titration of surfactant DMHNHC14 at 25ºC. The curve shows the fitting of the experimental points to the acid base equations including the presence of CO_2.

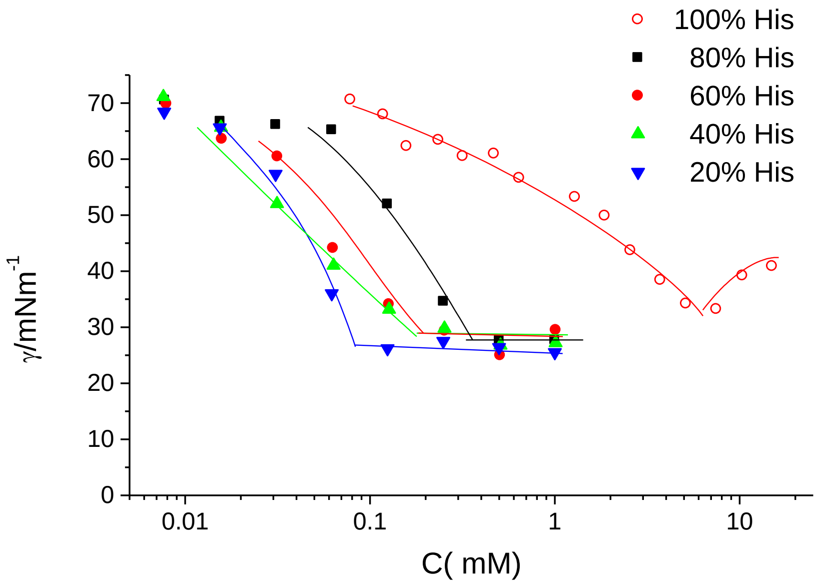

Figure S2. Surface tension as a function of concentration for mixtures of histidine surfactant with $C_{12}C_3L$ at 25 ℃

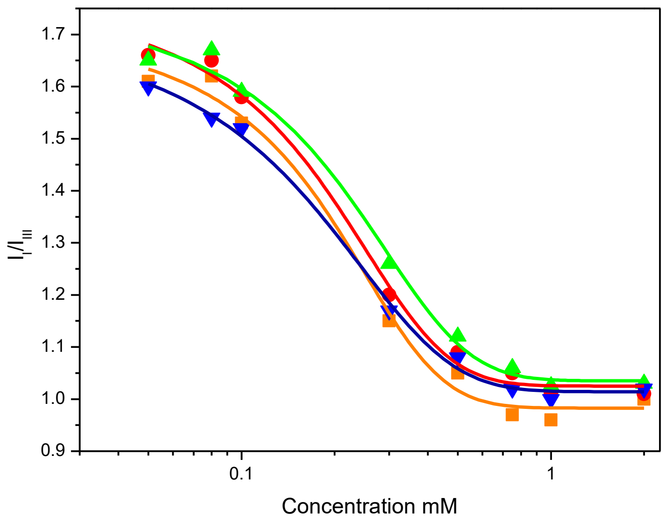

Figure S3. I1/I3 values for pyrene as a function of DMHNHC14/C3C12L catanionic mixtures concentration (■20:80HL ● 40:60 ▲60:80HL ▼80:20HL)

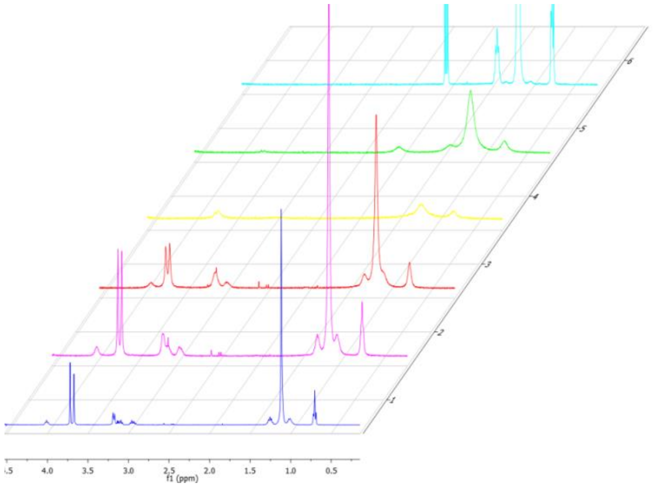

Figure S4. H^1NMR of the pure surfactants and the catanionic mixtures obtained from 5 mM solutions at 25 ℃ (1 Histidine, 2 80:20HM, 3 60:40HM, 4 40:60HM, 5 20:80HM, 6 Myristate)

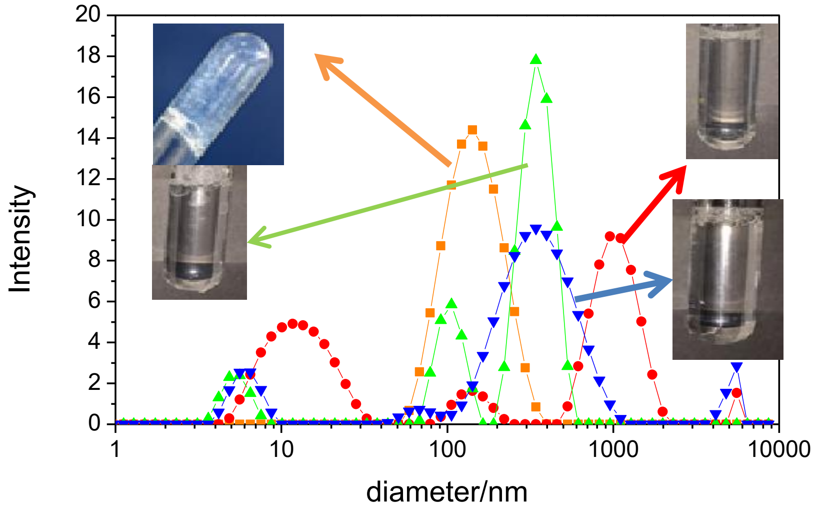

Figure S5.Intensity as a function of diameter as obtained from dynamic light scattering obtained at 25 ℃ for the different compositions of DMHNHC14/$C_3C_{12}L$ ▼80:20HL ▲60:80HL ● 40:60 ■20:80HL

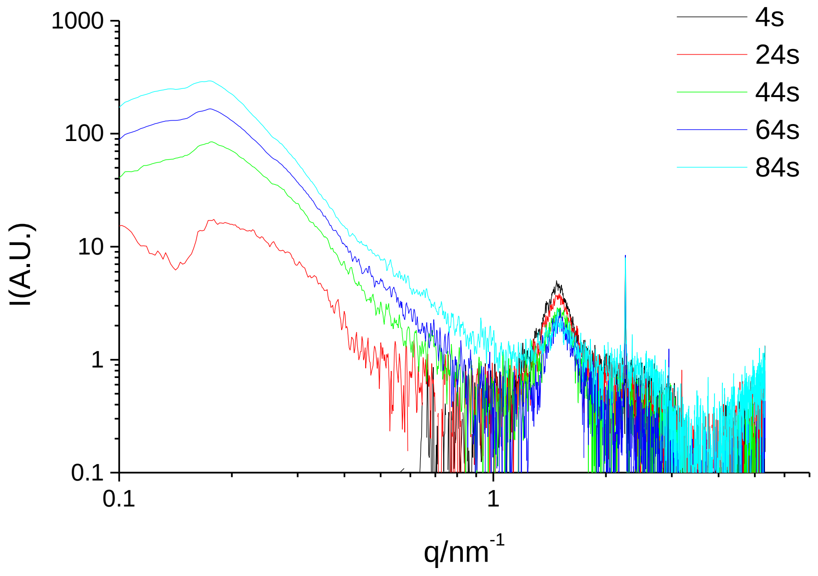

Figure S6. DMHNHC14/$C_3C_{12}L$ 80:20 HL SAXS intensity as a function of scattering vector q at different times. Note that the peak at 1=1.5 nm^{-1} decreases in intensity while the intensity at small q increases. The peak at q=2.25 nm^{-1} appears after 10 s measurement.

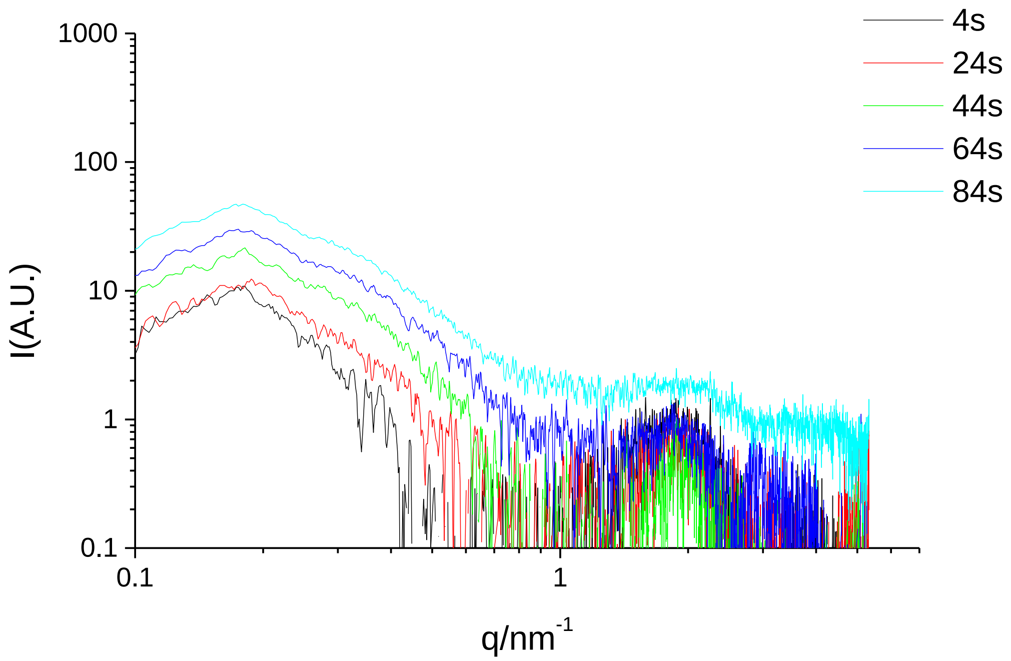

Figure S7. DMHNHC14/C_3C_{12}L 40:60 HL SAXS intensity as a function of scattering vector q at different times.. The overall intensity increases wiht time, the band around 2 nm^{-1} becomes less marked.

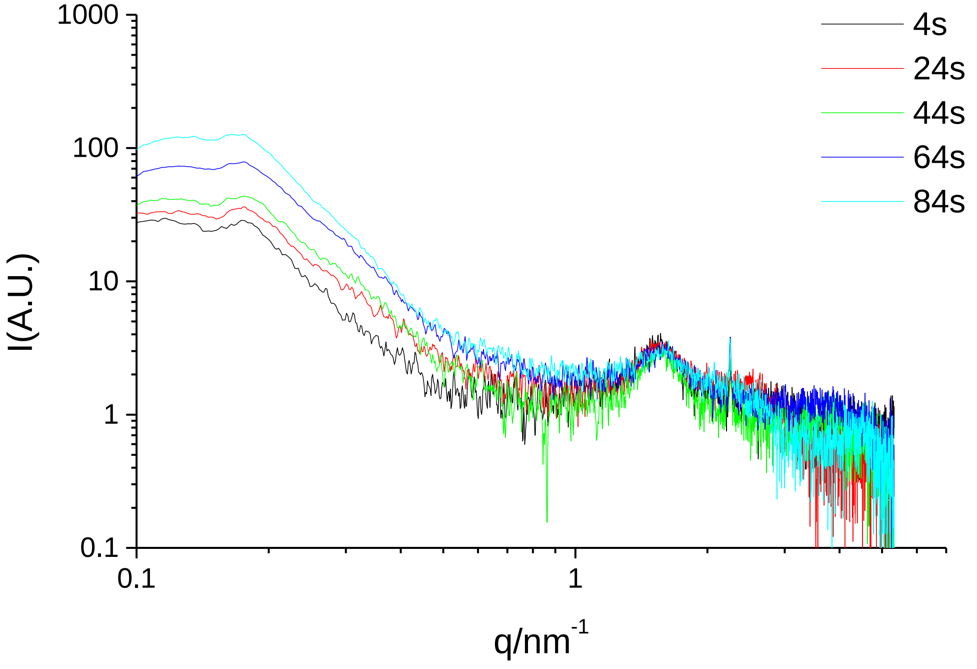

Figure S8. DMHNHC14/C_3C_{12}L 20:80 HL SAXS intensity as a function of scattering vector q at different times... The behaviour is similar to 80:20 HL but the peak at q= 2.25 nm^{-1} appears after 30 s.

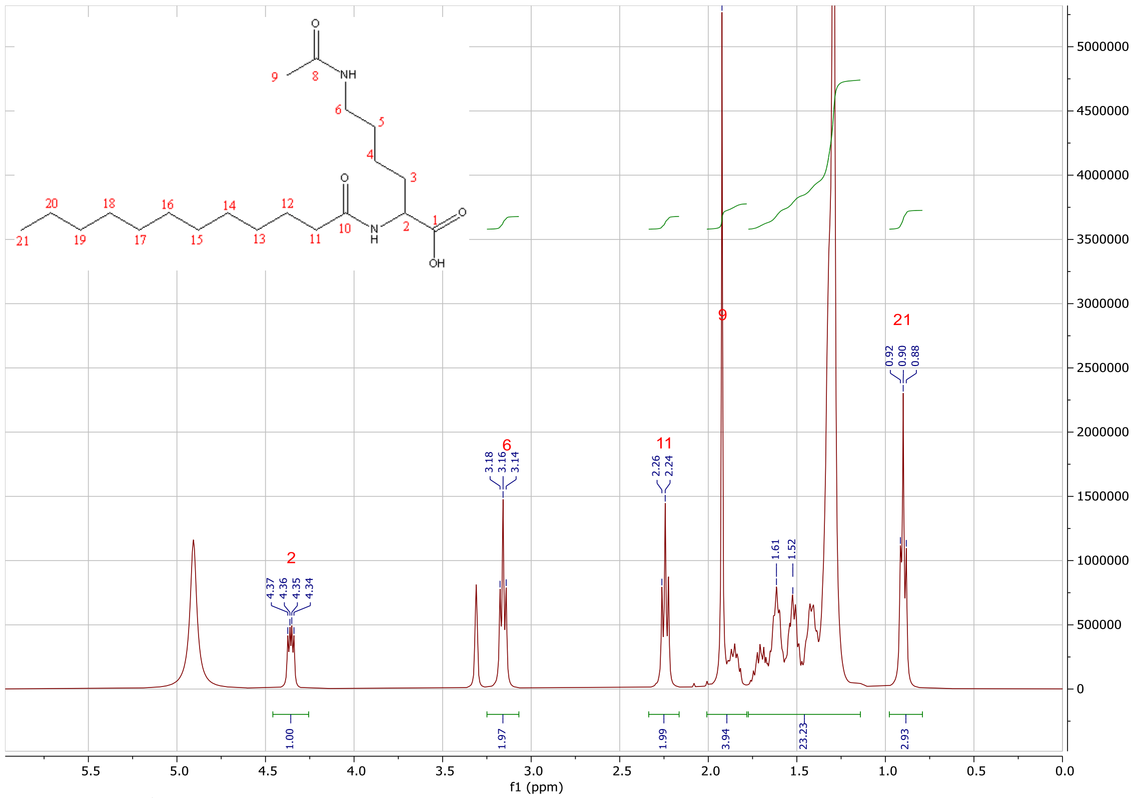

Figure S9. ^{1}HNMR spectrum of $C_{12}C_3L$

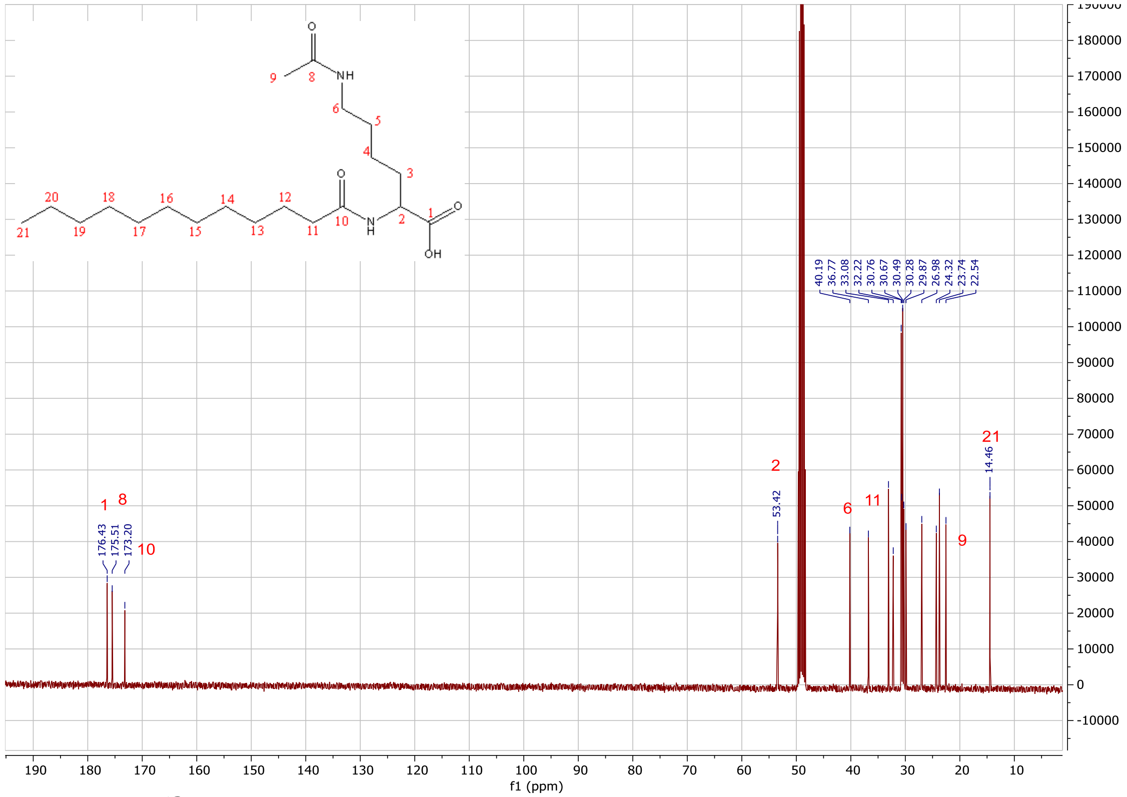

Figure S10. ^{13}CNMR spectrum of $C_{12}C_3L$

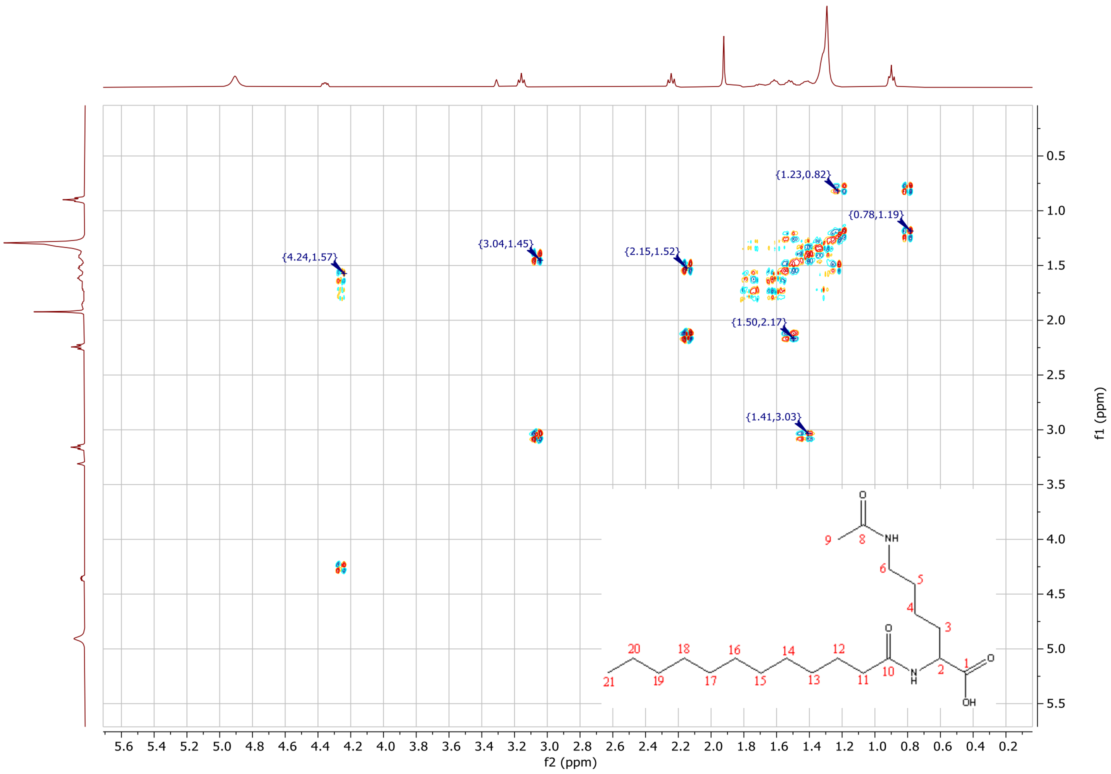

Figure S11. COSY spectrum of $C_{12}C_3L$

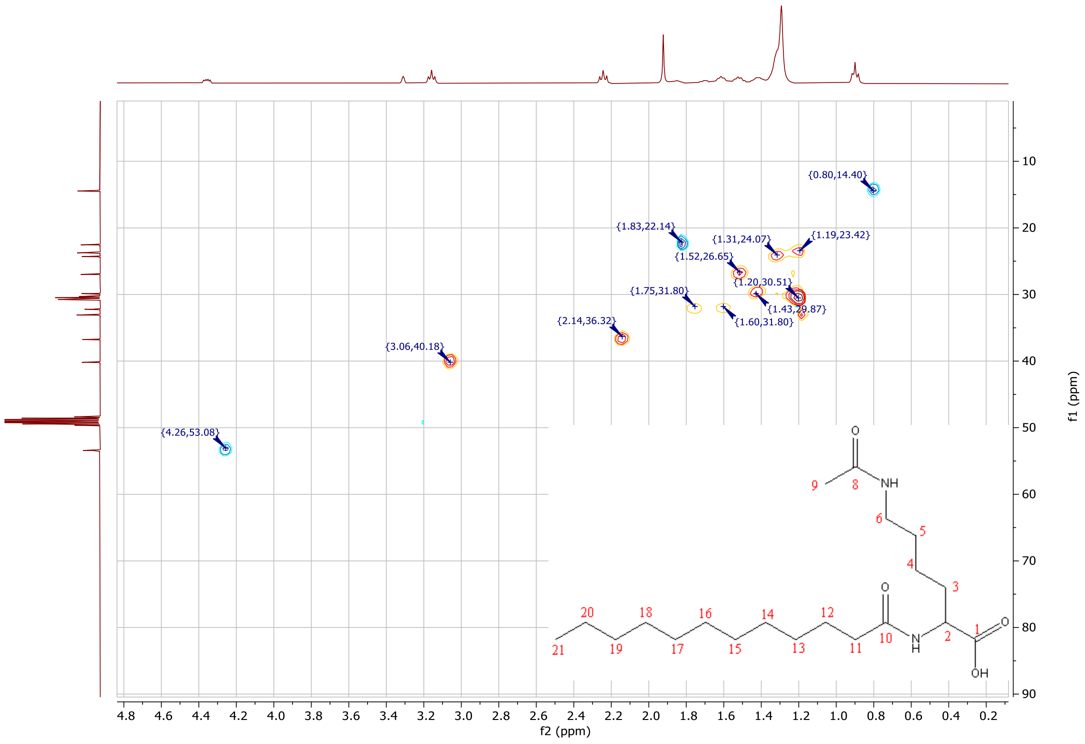

Figure S12. HSQC spectrum of $C_{12}C_3L$

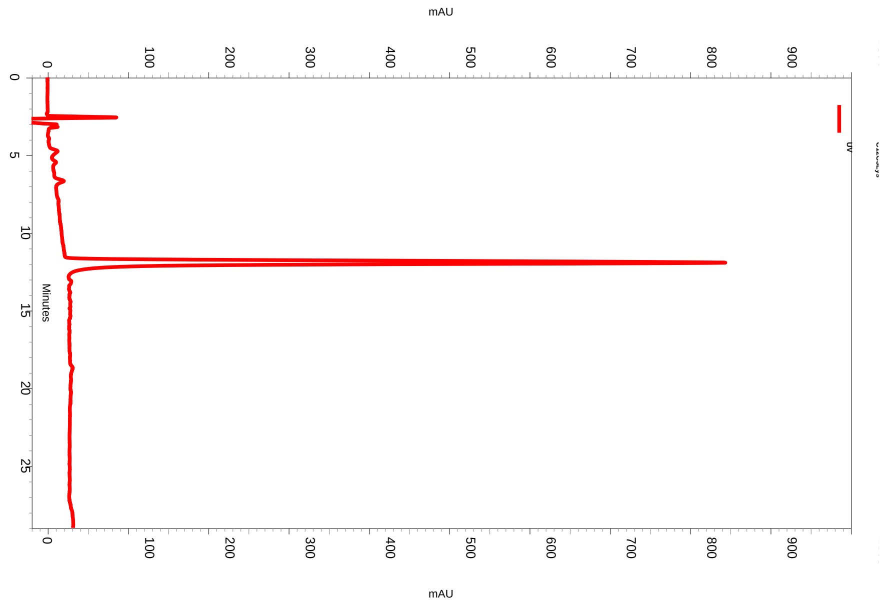

Figure S13. HPLC chromatogram of $C_{12}C_3L$

Table S1 Surface tension and fluorescence aggregation parameters of the pure surfactants and the catanionic mixtures and ζ-potential of the mixtures.

His%	α His[1]	A_m[2]	CMC_γ[3]	γ_{cmc}[4]	CMC_F[3]	ζ-potential[5]
100	1	57	5.2	33	3.8	
80:20HL	0.748	19	0.33	28	0.07	+41mV

60:40HL	0.527	22	0.19	28	0.15	+41mV
40:60HL	0.331	30	0.17	29.5	0.13	+41mV
20:80HL	0.157	14	0.09	26.5	0.15	~0

[1] Histidine molar fraction in the mixture.
[2] Area per molecule according to Gibbs adsorption isotherm using n=1 in nm^2.
[3] CMC obtained by surface tension or Fluorescence in mM.
[4] Surface tension at CMC in mNm^{-1}.
[5] ζ-potential in mV.
